# Report of the Committee of Distribution to the Council

**Published:** 1913-08-09

**Authors:** 


### REPORT OF THE COMMITTEE OF DISTRIBUTION TO THE COUNCIL.

The Committee of Distribution held their first meeting on Tuesday, May 20, and then and at subsequent meetings examined the reports and income and expenditure accounts of the several institutions seeking to participate, and made their awards in conformity with the Laws of the Constitution.

The Committee beg to report that the total of the Fund to August 5 amounts to £59,381.

The Fund has received a legacy of £20 from the estate of Mr. William Prior, deceased, and a further payment of £300 on account of the bequest of the late Mr. C. E. Layton.

This year 263 institutions have applied to participate in the Fund, being six more than in 1912, and the Committee now recommend the distribution of £59,134 to the several hospitals (except as below), institutions, dispensaries, and nursing associations as shown in the appendix hereto.

The Committee having reduced the bases of award to nine institutions, the governing bodies of the same were informed thereof, in accordance with the Laws of the Constitution. Five deputations attended the Committee, and, after hearing their several explanations, the bases of award were finally determined.

The Committee have determined the basis of award to St. George's Hospital, but they recommend that no order be made as to payment until the Committee is satisfied that no payment is made from the General Fund of the Hospital to the Medical School except for work done in the treatment of the patients.

The Committee have determined the basis of award to St. John's Hospital for Diseases of the Skin, but they recommend that no order be made as to payment pending the result of the litigation, which is still undecided.

Seven and a half per cent. of the total sum available for distribution is appropriated to the purchase of surgical appliances during the ensuing year, and 2½ per cent. for district nursing associations.

In compliance with an order of the Council, statistics have been prepared as usual for the special use of its members, showing the number of beds in hospitals, the cost of patients at hospitals and dispensaries, the proportionate expense of management to maintenance, and other valuable information. A copy thereof has been sent to each member of the Council.

David Burnett, Lord Mayor, President and Treasurer.

| | |
|---|---|
| David Anderson. | Robert Grey. |
| John C. Bell. | G. H. Makins. |
| F. A. Bevan. | Adrian Pollock. |
| William S. Church. | Marcus Samuel. |
| Savile Crossley. | E. Parker Young. |

Richard B. Martin, John Henry Buxton, } Hon. Secs.

Mansion House, July 23, 1913.

### Awards Recommended by the Committee of Distribution for the Year 1913.

THIRTY-THREE GENERAL HOSPITALS.

Charing Cross Hospital, £1,015 16s. 8d.; French Hospital, £341 13s. 4d.; German Hospital, £484 3s. 4d.; Great Northern Central Hospital, £1,234 3s. 4d.; Guy's Hospital, £1,630 16s. 8d.; Hampstead and N.W. London Hospital, £693 6s. 8d.; Italian Hospital, £209 3s. 4d.; Kensington General Hospital, £106 13s. 4d.; King's College Hospital, £1,442 10s.; London Hospital, £5,510; London Homœopathic Hospital, £437 10s.; Phillips Memorial Homœopathic Hospital, £46 13s. 4d.; London Temperance Hospital, £609 3s. 4d.; Metropolitan Hospital, £1,104 3s. 4d.; Mildmay Hospital, £213 6s. 8d.; Mildmay Memorial Hospital, £40 16s. 8d.; Miller Hospital and Royal Kent Dispensary, £325; Poplar Hospital, £544 3s. 4d.; Prince of Wales's General Hospital, Tottenham, £810 16s. 8d.; Royal Free Hospital, £1,270; *St. George's Hospital; SS. John and Elizabeth Hospital, £165; St. John's Hospital, Lewisham, £200 16s. 8d.; St. Mary's Hospital, £1,877 10s.; St. Thomas's Hospital, £345; Seamen's Hospital Society, £1,560 16s. 8d.; the Middlesex Hospital and Convalescent Home, £2,421 13s. 4d.; University College Hospital, £1,888 6s. 8d.; Walthamstow, etc., Hospital, £129 3s. 4d.; Wandsworth, Bolingbroke Hospital, £350; West Ham Hospital, £663 6s. 8d.; West London Hospital, £1,096 13s. 4d.; Westminster Hospital, £955.

SIX CHEST HOSPITALS.

City of London Hospital for Diseases of the Chest, Victoria Park, £994 3s. 4d.; Hospital for Consumption, Brompton, £2,468 6s. 8d.; Mount Vernon Consumption Hospital, Hampstead, £1,075; Royal Hospital for Diseases of the Chest, £495 16s. 8d.; Royal National Sanatorium, £66 13s. 4d.; Royal National Hospital for Consumption, £208 6s. 8d.

TWENTY CHILDREN'S HOSPITALS.

Alexandra Hospital for Hip Disease, £294 3s. 4d.; Banstead Surgical Home, £15 16s. 8d.; Barnet Home Hospital, £35 16s. 8d.; Belgrave Hospital for Children, £179 3s. 4d.; Cheyne Hospital for Incurable Children, £25; East London Hospital for Children, £785; Evelina Hospital for Sick Children, £123 6s. 8d.; Home for Incurable Children, £46 13s. 4d.; Home for Sick Children, Sydenham, £103 6s. 8d.; Hospital for Sick Children, £1,185 16s. 8d.; Infants' Hospital, Westminster, £195 16s. 8d.; Kensington Hospital for Children and Dispensary, £76 13s. 4d.; Paddington Green Hospital for Children, £217 10s.; Queen's Hospital for Children, £1,010; Royal Waterloo Hospital for Children and Women, £434 3s. 4d.; St. Mary's Hospital, Plaistow, £226 13s. 4d.; St. Monica's Hospital, Brondesbury, £63 6s. 8d.; Victoria Hospital for Children, Chelsea, £619 3s. 4d.; Victoria Home, Margate, £35; Hospital for Hip Disease, Sevenoaks, £54 3s. 4d.

* See Report of Distribution Committee.

### EIGHT LYING-IN HOSPITALS.

British Lying-in Hospital, Endell Street, £120 16s. 8d.; City of London Lying-in Hospital, £180 16s. 8d.; Clapham Maternity Hospital and Dispensary, £25 16s. 8d.; East End Mothers' Home, £75; General Lying-in Hospital, Lambeth, £190 16s. 8d.; Plaistow Maternity Charity, £55; Queen Charlotte's Lying-in Hospital, £510 16s. 8d.; Home for Mothers and Babies, Woolwich, £45 16s. 8d.

### FIVE HOSPITALS FOR WOMEN.

Chelsea Hospital for Women, £283 6s. 8d.; Hospital for Women, Soho Square, £333 6s. 8d.; Grosvenor Hospital for Women and Children, £122 10s.; New Hospital for Women, £288 6s. 8d.; Samaritan Free Hospital, £377 10s.

### TWENTY-FIVE OTHER SPECIAL HOSPITALS.

Middlesex Hospital, Cancer Wing, £216 13s. 4d.; London Fever Hospital, Islington, £250; Gordon Hospital for Fistula, £39 3s. 4d.; St. Mark's Hospital for Fistula, £296 13s. 4d.; National Hospital for the Diseases of the Heart, £91 13s. 4d.; Female Lock Hospital, Harrow Road, £305 16s. 8d.; Hospital for Epilepsy, Paralysis, and other Diseases of the Nervous System, £188 6s. 8d.; National Hospital for the Paralysed and Epileptic, £816 13s. 4d.; West End Hospital for Diseases of the Nervous System, £360 16s. 8d.; Central London Ophthalmic Hospital, £129 3s. 4d.; Royal Eye Hospital, £244 3s. 4d.; Royal London Ophthalmic Hospital, £818 6s. 8d.; Royal Westminster Ophthalmic Hospital, £136 13s. 4d.; Western Ophthalmic Hospital, £68 6s. 8d.; Royal National Orthopædic Hospital, £502 10s.; Royal Sea Bathing Hospital, Margate, £137 10s.; *St. John's Hospital for Diseases of the Skin; St. Peter's Hospital for Stone, £120 16s. 8d.; Central London Throat and Ear Hospital, £14 3s. 4d.; Throat Hospital, Golden Square, £38 6s. 8d.; London Throat Hospital, £30 16s. 8d.; Metropolitan Ear, Nose, and Throat Hospital, £20 16s. 8d.; Royal Ear Hospital, Dean Street, £64 3s. 4d.; Royal Dental Hospital of London, £177 10s.; National Dental Hospital, £54 3s. 4d.

### THIRTY-FOUR CONVALESCENT HOSPITALS.

Metropolitan Convalescent Institution, Walton, £309 3s. 4d.; Metropolitan Convalescent Institution, Broadstairs, £211 13s. 4d.; Metropolitan Convalescent Institution, Bexhill, £420; All Saints' Convalescent Hospital, Eastbourne, £333 6s. 8d.; All Saints' Convalescent Home, St. Leonards, £20 16s. 8d.; Ascot Priory Convalescent Home, £70 16s. 8d.; Brentwood Convalescent Home for Children, £14 3s. 4d.; Charing Cross Hospital Convalescent Home, £54 3s. 4d.; Chelsea Hospital for Women, Convalescent Home, St. Leonards, £50; Deptford Medical Mission Convalescent Home, £15 16s. 8d.; Mrs. Gladstone's Convalescent Home, Mitcham, £65 16s. 8d.; Friendly Societies' Convalescent Home, Dover, £83 6s. 8d.; Hahnemann Convalescent Home, Bournemouth, £25; Hanwell Convalescent Home, £8 6s. 8d.; Hastings, Fairlight Convalescent Home, £53 6s. 8d.; Hendon, Ossulston Home, £39 3s. 4d.; Herbert Convalescent Home, Bournemouth, £14 3s. 4d.; Herne Bay Baldwin-Brown Convalescent Home, £50; Homœopathic Hospital Convalescent Home, £5; Hemel Hempstead Convalescent Home, £20 16s. 8d.; Mrs. Kitto's Convalescent Home, Reigate, £40 16s. 8d.; London Hospital Convalescent Home, Tankerton, £58 6s. 8d.; Mary Wardell Convalescent Home for Scarlet Fever, £41 13s. 4d.; Police Seaside Home, Brighton, £40; St. Andrew's Convalescent Home, Clewer, £75; St. Andrew's Convalescent Home, Folkestone, £133 6s. 8d.; St. John's Home for Convalescent and Crippled Children, £21 13s. 4d.; St. Joseph's Convalescent Home, Bournemouth, £25; St. Leonards-on-Sea Convalescent Home for Poor Children, £83 6s. 8d.; Eversfield, St. Leonard's, £29 3s. 4d.; St. Mary's Convalescent Home, Broadstairs, £125; St. Mary's Convalescent Home, Shortlands, £15; St. Michael's Convalescent Home, Westgate-on-Sea, £27 10s.; Seaside Convalescent Hospital, Seaford, £70 16s. 8d.

* See Report of Distribution Committee.

### TWENTY-FOUR COTTAGE HOSPITALS.

Acton Cottage Hospital, £113 6s. 8d.; Beckenham Cottage Hospital, £75; Blackheath and Charlton Cottage Hospital, £75; Bromley, Kent, Cottage Hospital, £88 6s. 8d.; Bushey Heath Cottage Hospital, £27 10s.; Canning Town Cottage Hospital, £78 6s. 8d.; Chislehurst, Sidcup, and Cray Cottage Hospital, £58 6s. 8d.; Coldash Cottage Hospital, £41 13s. 4d.; Ealing Cottage Hospital, £111 13s. 4d.; East Ham Cottage Hospital, £55; Eltham Cottage Hospital, £54 3s. 4d.; Enfield Cottage Hospital, £74 3s. 4d.; Epsom and Ewell Cottage Hospital, £40 16s. 8d.; Hounslow Cottage Hospital, £37 10s.; Livingstone Dartford Cottage Hospital, £91 13s. 4d.; Kingston, Victoria Hospital, £55 16s. 8d.; Reigate and Redhill Hospital, £136 13s. 4d.; Sidcup Cottage Hospital, £30 16s. 8d.; Teddington, £80; Tilbury (Passmore Edwards) Cottage Hospital, £54 3s. 4d.; Willesden Cottage Hospital, £90; Wimbledon Cottage Hospital, £51 13s. 4d.; Wimbledon (South) Cottage Hospital, £47 10s.; Wood Green Cottage Hospital, £70 16s. 8d.

### TWELVE INSTITUTIONS.

Hospital for Invalid Gentlewomen, £85 16s. 8d.; St. Saviour's Hospital and Nursing Home, £70 16s. 8d.; Invalid Asylum, Stoke Newington, £26 13s. 4d.; Firs Home, Bournemouth, £29 3s. 4d.; Friedenheim Hospital for the Dying, £204 3s. 4d.; Free Home for Dying, Clapham, £140 16s. 8d.; St. Luke's House, Pembridge Square, £131 13s. 4d.; Santa Claus Home, Highgate, £60; Royal Mineral Water Hospital, Bath, £50; Winifred House, Holloway, £50; Highgate Convalescent Home, £33 6s. 8d.; Erskine House, Hampstead, £27 10s.

### FIFTY-SEVEN DISPENSARIES.

Battersea Provident Dispensary, £183 6s. 8d.; Billingsgate Dispensary, £45; Blackfriars Provident Dispensary, £15; Bloomsbury Provident Dispensary, £12 10s.; Brixton, etc., Dispensary, £40 16s. 8d.; Brompton Provident Dispensary, £15; Buxton Street Dispensary, £10 16s. 8d.; Camberwell Provident Dispensary, £56 13s. 4d.; Camden Town Provident Dispensary, £12 10s.; Chelsea Provident Dispensary, £10 16s. 8d.; Child's Hill Provident Dispensary, £7 10s.; City Dispensary, £58 6s. 8d.; Clapham General and Provident Dispensary, £19 3s. 4d.; Deptford Medical Mission, £21 13s. 4d.; Eastern Dispensary, £30; East Dulwich Provident Dispensary, £54 3s. 4d.; Farringdon General Dispensary, £34 3s. 4d.; Finsbury Dispensary, £40; Forest Hill Provident Dispensary, £32 10s.; Greenwich Provident Dispensary, £24 3s. 4d.; Hackney Provident Dispensary, £18 6s. 8d.; Hampstead Provident Dispensary, £39 3s. 4d.; Holloway and North Islington Dispensary, £12 10s.; Islington Dispensary, £40; Islington Medical Mission, £42 10s.; Kennington and Vauxhall Provident Dispensary, £11 13s. 4d.; Kensal Town Provident Dispensary, £11 13s. 4d.; Kentish Town

Medical Mission, £19 3s. 4d.; Kilburn, Maida Vale, and St. John's Wood Dispensary, £29 3s. 4d.; Kilburn Provident Medical Institution, £41 13s. 4d.; London Dispensary, Spitalfields, £11 13s. 4d.; London Medical Mission, Endell Street, £70 16s. 8d.; Margaret Street, for Consumption, £23 6s. 8d.; Metropolitan Dispensary, £42 10s.; Mildmay Medical Mission Dispensary, £8 6s. 8d.; Notting Hill Provident Dispensary, £18 6s. 8d.; Paddington Provident Dispensary, £29 3s. 4d.; Public Dispensary, Drury Lane, W.C., £33 6s. 8d.; Queen Adelaide's Dispensary, £19 3s. 4d.; Royal General Dispensary, £22 10s.; Royal Pimlico Provident Dispensary, £26 13s. 4d.; Royal South London Dispensary, £39 3s. 4d.; St. George's, Hanover Square, Dispensary, £28 6s. 8d.; St. John's Wood Provident Dispensary, £39 3s. 4d.; St. Marylebone General Dispensary, £54 3s. 4d.; St. Pancras and Northern Dispensary, £31 13s. 4d.; South Lambeth, Stockwell, and North Brixton Dispensary, £18 6s. 8d.; Stamford Hill, Stoke Newington Dispensary, £46 13s. 4d.; Tower Hamlets Dispensary, £32 10s.; Walworth Provident Dispensary, £12 10s.; Wandsworth Common Provident Dispensary, £8 6s. 8d.; Westbourne Provident Dispensary, £15; Western Dispensary, £61 13s. 4d.; Western General Dispensary, £70; Westminster General Dispensary, £35; Whitechapel Provident Dispensary, £26 13s. 4d.; Woolwich Provident Dispensary, £31 13s. 4d.

THIRTY-THREE NURSING ASSOCIATIONS.

Belvedere, Abbey Wood, £6 6s.; Brixton, £25 4s.; Central St. Pancras, £25 4s.; Chelsea and Pimlico, £25 4s.; Hackney, £25 4s.; Hammersmith, £50 8s.; Hampstead, £18 18s.; Isleworth, £12 12s.; Kensington, £50 8s.; Kilburn, £6 6s.; Kingston, £44 2s.; Lambeth Road (Catholic), £12 12s.; Metropolitan (Bloomsbury), £44 2s.; St. Olave's (Bermondsey), £18 18s.; Paddington and Marylebone, £44 2s.; Peckham, £12 12s.; Plaistow, £138 12s.; Plaistow (Maternity), £176 8s.; Ponders End, Enfield, etc., £12 12s.; Rotherhithe, £12 12s.; Shoreditch, £50 8s.; Sick Room Helps Society, £18 18s.; Sidcup, £6 6s.; Silvertown, £18 18s.; South London (Battersea), £63; Southwark, £50 8s.; South Wimbledon, £31 10s.; Tottenham, £18 18s.; Westminster, £25 4s.; Woolwich, £25 4s.; East London, £144 18s.; North London, £63; London District, £390 12s.

The Council are prepared to receive and distribute as part of the annual receipts, special gifts or bequests from any who may wish to assist the hospitals of London and are in doubt as to those most deserving their bounty.

Grants are made only to institutions furnishing the Committee of Distribution with proofs of their usefulness and efficiency, and properly audited accounts.

Form of Bequest.

I give and bequeath to the Metropolitan Hospital Sunday Fund the sum of........................ (free of legacy duty) to be applied to the purposes of the Fund, and the receipt of the Treasurer for the time being of the said Fund shall be a sufficient discharge for the same.

